# Personal

**Published:** 1910-06

**Authors:** 


### PERSONAL.

Dr. J. F. Fairbairn, of Buffalo, announces that the removal of his offices was made on May 1, to the Professional Building, corner Allen Street and Irving Place. Practice confined to ear, nose and throat. Hours: 9 a. m. to 1 p. m., and by appointment.

Dr. James A. Gardner, of Buffalo, whose office is at 403 Franklin Street, announces hereafter that his office hours will be held in the morning from 10 a. m. until 12 o'clock.

Dr. L. Schroeter, of Buffalo, has removed to 415 Franklin Street, between Virginia and Edward Streets. Hours: 9 to 10 a. m., and 7 to 8 p. m. Telephones: Bell, Tupper 747; Frontier, 73. Branch office: 798 Fillmore Avenue. Hours: 1 to 3 p. m.

Dr. Clayton K. Haskell, of Bath, N. Y., has been granted two months' leave of absence from his position as chief surgeon of the hospital at the Soldiers' Home, and will spend the time in a postgraduate course in surgical work at Johns Hopkins University. During his absence Dr. Raymond C. Hill of Sodus, first assistant surgeon, will be in charge of the hospital.

Dr. and Mrs. Carlton R. Jewett, of Buffalo, are contemplating a European trip in July and will remain about six weeks abroad.

Brevet Lieutenant Colonel A. H. Briggs, M. D., Major Surgeon of the 65th Regiment, N. G., N. Y., has been promoted to be Chief Medical Officer of the Fourth Brigade. In celebration of the event, Colonel Briggs reviewed the brigade Tuesday evening, April 26, 1910. A concert preceded the review and dancing followed, the whole evening being up to the honors of the new brigade surgeon.

Dr. H. H. Glosser, of Buffalo, has removed his office from 516 South Park Avenue to 131 Allen Street, Professional Building. Hours from 9 a. m. to 1 o'clock.

---

Dr. Nelson G. Russell, of Buffalo, has been promoted to be surgeon with the rank of major, of the 65th Regiment, N. G., N. Y., Vice Brevet Lieutenant Colonel A. H. Briggs, promoted to be brigade surgeon.

---

Dr. A. W. Hengerer, of Buffalo, has removed his office and residence from 301 E. Genesee to 441 Pratt Street, near Genesee. Hours: 8 to 9 a. m., 1 to 2 and 7 p. m. Sundays: 2 to 4 only. Both telephones.

---

Dr. Harvey W. Wiley, of Washington, chief of the bureau of chemistry of the Department of Agriculture, was elected president of the United States Pharmacopeial convention, to serve a term of ten years, at the session May 11, 1910. This may be regarded as a public recognition of Dr. Wiley's work for the higher standardisation of food and drugs.

---

Dr. Carroll J. Roberts, of Buffalo, has been promoted to Captain and Assistant Surgeon, 65th Regiment N. G., N. Y., vice Dr. N. G. Russell promoted to be surgeon with the rank of Major.

---

Dr. William H. Park, of New York, professor of bacteriology and hygiene in the University and Bellevue Hospital Medical College, director of the bacteriological laboratory of the New York board of health, state medical examiner in hygiene and sanitation, has received the degree of LL.D., from Queen's University, Kingston, Ontario, Canada.

---

Dr. William T. Getman, of Buffalo, has been appointed assistant surgeon of the 65th Regiment N. G., N. Y., with the rank of first lieutenant, vice Dr. Carroll J. Roberts, promoted.

---

Dr. J. N. McCormack, of Bowling Green, Ky., delivered an address at a dinner given by the Board of Trade of North Tonawanda, N. Y., at the Hotel Sheldon, May 23, 1910. He spoke also at Lockport and Niagara Falls on the two following days. The subject of his addresses being "The purification of the water of the Niagara River." Owing to the prevalence of typhoid fever at Niagara Falls, much interest is manifested on this subject. Dr. McCormick came on the invitation of Honorable J. S.

Simmons, representative in congress from the thirty-fourth district.

---

Dr. Charles Gordon Heyd, formerly of Buffalo, now of the New York Post Graduate Medical School and Hospital was the guest of his uncle, Dr. H. E. Hayd, during commencement week at the University.

